# Temporal variations in the pattern of breathing: techniques, sources, and applications to translational sciences

**DOI:** 10.1186/s12576-022-00847-z

**Published:** 2022-08-29

**Authors:** Yoshitaka Oku

**Affiliations:** Division of Physiome, Department of Physiology, Hyogo Medical University, Nishinomiya, Hyogo 663-8501 Japan

**Keywords:** Complex variability, Emotion, Cognition, Coupled oscillators, Cross-frequency coupling, Nonlinear analysis

## Abstract

The breathing process possesses a complex variability caused in part by the respiratory central pattern generator in the brainstem; however, it also arises from chemical and mechanical feedback control loops, network reorganization and network sharing with nonrespiratory motor acts, as well as inputs from cortical and subcortical systems. The notion that respiratory fluctuations contain hidden information has prompted scientists to decipher respiratory signals to better understand the fundamental mechanisms of respiratory pattern generation, interactions with emotion, influences on the cortical neuronal networks associated with cognition, and changes in variability in healthy and disease-carrying individuals. Respiration can be used to express and control emotion. Furthermore, respiration appears to organize brain-wide network oscillations via cross-frequency coupling, optimizing cognitive performance. With the aid of information theory-based techniques and machine learning, the hidden information can be translated into a form usable in clinical practice for diagnosis, emotion recognition, and mental conditioning.

## Introduction

Humans breathe throughout their lives; from birth to death, our breaths are always changing, and no single breath is identical to another. Where do these variations originate from? What information can we extract from the variations in breathing? What are the physiological and pathophysiological implications of these variations? Breath-to-breath variations in breathing patterns can occur as uncorrelated random variations (white noise), correlated random changes, periodic variations, or nonrandom, nonperiodic fluctuations [1]. This review focuses on these temporal variations in the pattern of breathing, including the complex variability of breathing and characteristic breathing patterns.

*Definition of terms* The term “variability” is used in different ways. For example, heart rate variability refers to small fluctuations in the time interval between heartbeats. However, the term is also used to describe more diverse variations in the pattern of breathing. In this review, the term “respiratory variability” is used in a broader sense, as equivalent to “breath-to-breath variations in breathing patterns” described by Bruce [1]. The term “complexity”, synonymous with “complex variability”, has a meaning that is qualitatively and quantitatively distinguishable from traditional concepts and metrics of “variability” [2]. Although no formal definition of this term exists, it may be said that “complexity” is a measure of the amount of information and unpredictability, which is quantified with the information theory-based techniques mentioned in "Techniques for analyzing respiratory variability" section. As revealed in "Respiratory variability in health and disease" and "Future directions in translational sciences" sections, the concept of complex variability can be usefully applied to translational sciences. The term “nonlinearity” refers to the relationship between system inputs and outputs where the latter is not directly proportional to the former; thus, small input perturbations can cause large effects on the outputs [2]. A linear system, but not a nonlinear system, can be described by an autoregressive model or frequency response characteristics.

Recognizing the presence of 'hidden information' in physiological time series necessitates the use of fluctuation analysis techniques in statistical physics [3–7], which are unfamiliar to physiologists. Therefore, this review begins by summarizing techniques for analyzing complex variability and then discusses the sources of respiratory variability and translations of the information hidden therein to health-related sciences.

## Techniques for analyzing respiratory variability

A common measure of gross variability is the coefficient of variation (CV), which is defined as the ratio of the standard deviation to the mean. When datasets with varied units or considerably different means are compared, the CV should be used instead of the standard deviation. The root mean square successive difference (RMSSD) measures the extent of variability between successive time points; for example, the RMSSD of the interheartbeat interval can be used as an index for monitoring changes in parasympathetic activity [8].

Respiratory variability is produced through an integrated process that involves multifunctional control mechanisms in the brain; therefore, the characteristics of present breaths are correlated with the characteristics of past breaths. The autocorrelation (AR), which is defined as the correlation between a signal and a delayed copy of itself as a function of the delay, is a common correlation metric for discrete time-series data. For example, the white noise exhibits zero correlation with any nonzero time lag. On the other hand, if a signal is not random, one or more of the autocorrelations remains significantly nonzero. Previous studies on respiratory variability have typically used the AR at one breath lag [9].

In well-controlled experimental settings, respiratory control systems can be regarded as stationary. In such cases, the mean, standard deviation, CV, and AR remain invariant throughout the observation period. However, this is not always the case; in particular, during long-term observations, e.g., overnight monitoring, respiratory control systems become nonstationary. *Detrended fluctuation analysis* is a scaling analysis approach that was originally designed to quantify long-range power-law correlations in signals; however, it can also be used to investigate both long-term (LTCs) and short-term correlations (STCs) in nonstationary systems [10, 11]. Detrended fluctuation analysis uses the integrated fluctuation of a signal; the integrated time series is divided into *N* epochs of length *n*, and each epoch is detrended with a least squares fit, yielding a locally detrended time-series segment ${x}_{k}^{n}(t), k=1, \dots ,$ *N*. The average fluctuation for a given epoch is calculated as

$$F\left(n\right)=\sqrt{\frac{1}{N}\sum_{k=1}^{N}\sum_{t=1}^{n}{{x}_{k}^{n}(t)}^{2}}$$

The LTCs or STCs can then be extracted by the *scaling exponent*, α, which is the slope of $\frac{\mathrm{log}(F\left(n\right))}{\mathrm{log}(n)}$ for a specific range of *n* [12].

**Fig. 1:**
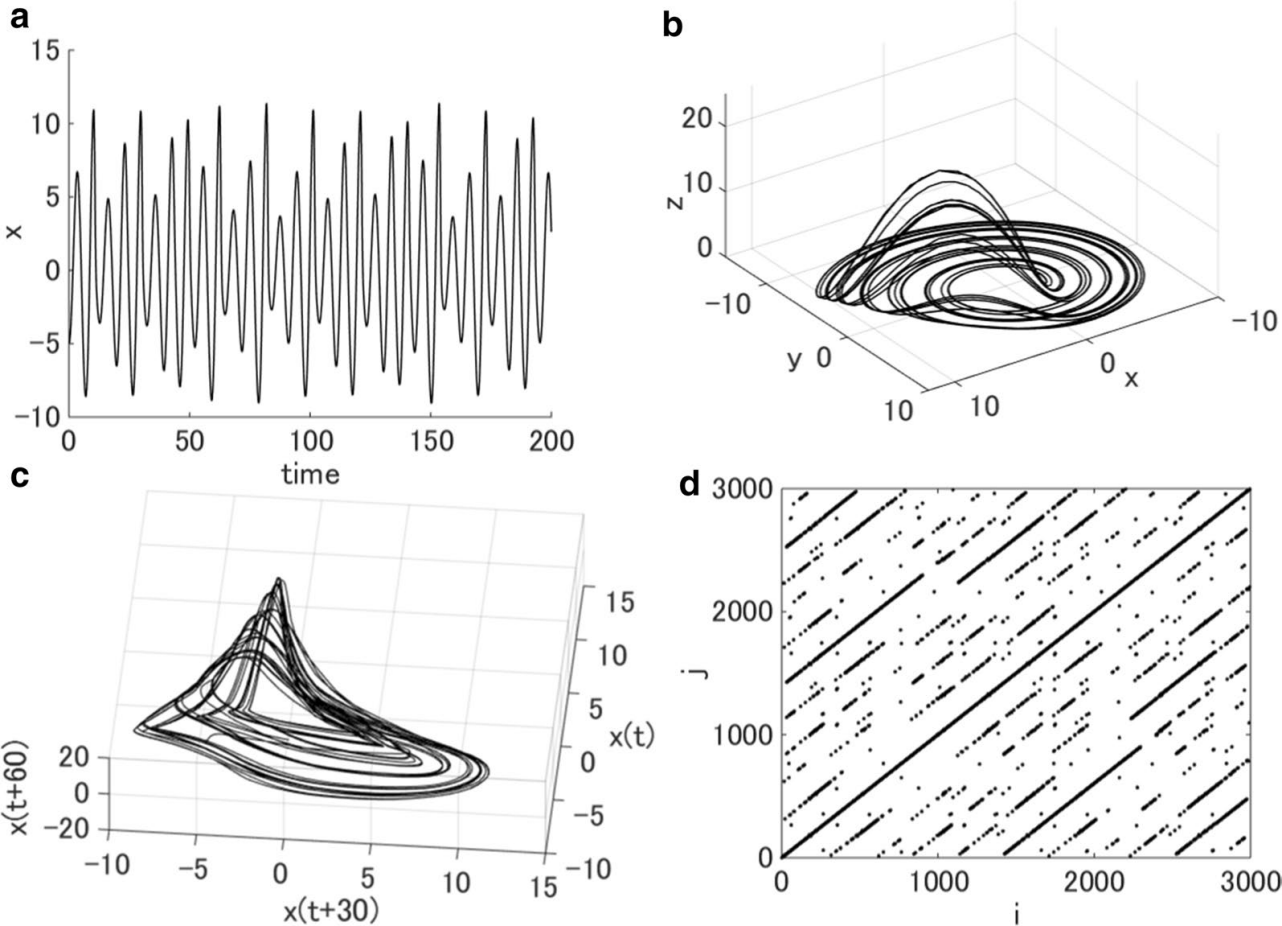
Visualization of the Rössler attractor, a continuous-time dynamical system that exhibits chaotic dynamics with fractal properties. The system is composed of three nonlinear differential equations and thus has three variables. **a** Temporal fluctuations of the x variable. Although each variable oscillates within a fixed range of values, its amplitude is highly variable. **b** Three-dimensional trajectory of the Rössler attractor visualized using the values of all three variables. **c** Three-dimensional plot of the Rössler attractor reconstructed using delay-time embedding ($\tau =30\,\mathrm{ a}.\mathrm{u}.$) of a single variable (x). Note that the complexity of the system is well preserved compared to **b,** in which all three variables are used. **d** Recurrence plot of the reconstructed attractor using delay-time embedding ($m=3, \tau =30\,\mathrm{ a}.\mathrm{u}.$, *r* = 1.0) consisting of diagonal lines with different lengths, indicating that the trajectories pass neighborhoods of past trajectories with limited durations. This means that the reconstructed attractor exhibits a fractal structure or self-similarity

Deterministic dynamical systems that follow a unique path or evolution can exhibit complex behaviors. One example is the Rössler attractor (Fig. 1), which is a system composed of three nonlinear ordinary differential equations. In such a system, a small difference in initial conditions can result in significantly different behaviors, and predicting future behavior becomes progressively more difficult with time, a traditional indicator of ‘chaos’. The *largest Lyapunov exponent* (LLE) measures the predictability of a system’s behavior; a positive LLE indicates that the attractor diverges, i.e., chaotic behavior [13].

The complexity of the variability of a system can be visualized through *state space reconstructions*. These visualizations can be obtained from a single observable variable using delay-time embedding (Fig. 1c). If we have time-series data $u\left(i\right),i=1, 2,\dots ,N$, the state space can be reconstructed as an m-dimensional state, ${X}_{i}^{m}=\{u\left(i\right), u\left(i+\tau \right), \dots , u\left(i+(m-1)\tau \right)\}$, where *m* is the embedding dimension and $\tau$ is the time delay. If we choose *m* and $\tau$ properly, then the complexity of the variability is well characterized by the reconstructed attractor [14, 15].

As seen in three-dimensional plots of the Rössler attractor (Fig. 1b), the trajectories never return to past trajectories but do pass near them. When we plot the neighborhoods ${X}_{j}$ of ${X}_{i}$ whose norm is within $r$, we can visualize these state recurrences [16]. This is called a *recurrence plot*, and it is mapped as

$${R}_{ij}=\Theta \left(r-\Vert {X}_{i}^{m}-{X}_{j}^{m}\Vert \right)$$

where $\Vert \cdot \Vert$ is a norm, $r$ is a threshold distance, and $\Theta$ is the Heaviside function, which has a value of 1 if the expression in the inner parenthesis has a value greater than zero and a value of zero otherwise. Diagonal lines of different lengths that appear in the recurrence plots (Fig. 1d) indicate that a state visits the same region of the attractor at different times [17]. Eckmann et al. [16] showed that the length of these short upward diagonal lines is inversely proportional to the LLE. Based on the probability of diagonal lines with specific lengths appearing, the amount of information or uncertainty can be calculated using an entropy measure, the *Shannon entropy* (ShEn) [17], which is formulated as

$$\mathrm{ShEn}= -\sum_{i=1}^{n}P\left({L}_{i}\right)\mathrm{log}P\left({L}_{i}\right)$$

where $P\left({L}_{i}\right) is$ is the probability that diagonal lines of length ${L}_{i}$ appear.

**Fig. 2:**
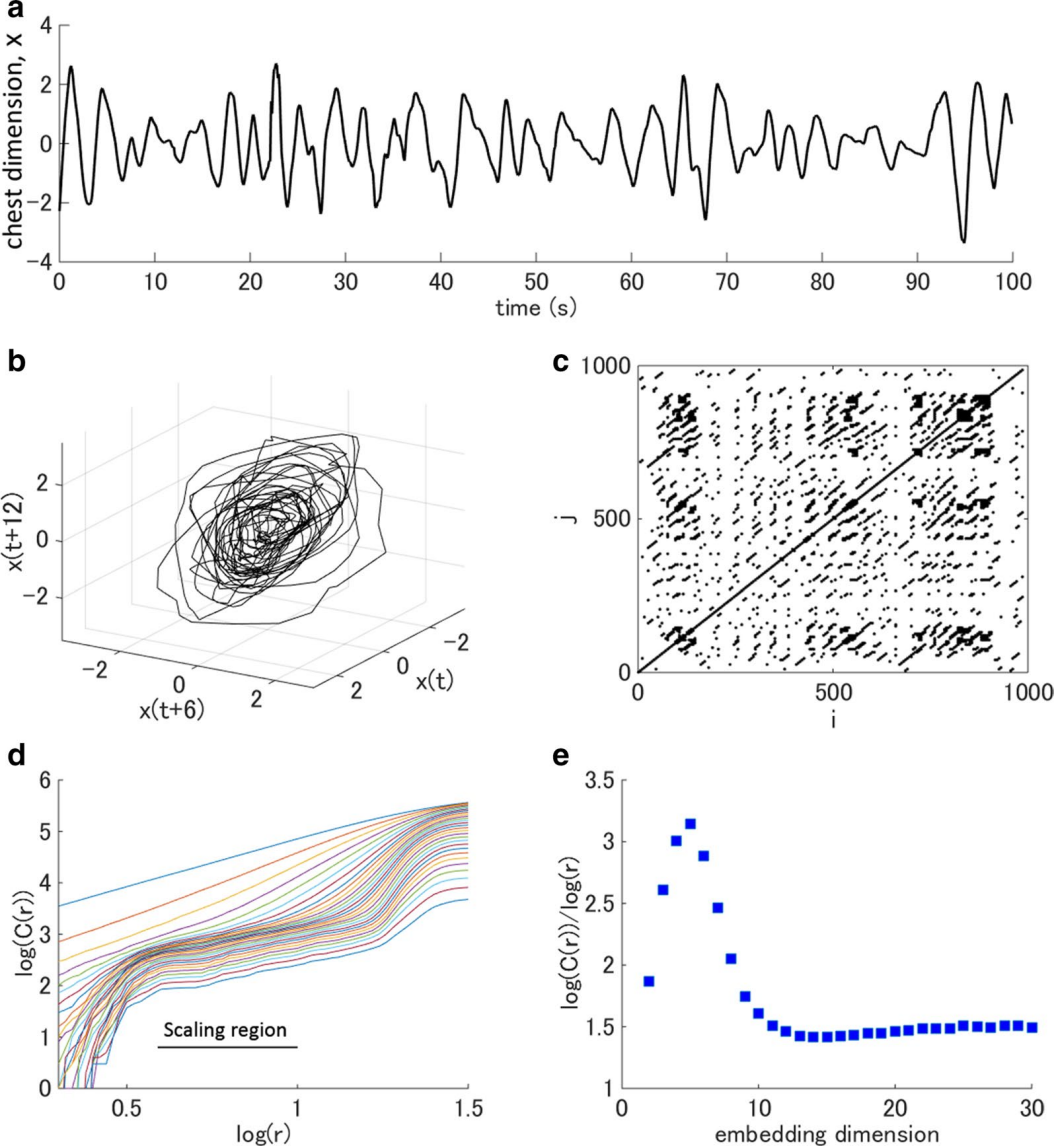
Visualization and quantification of the complexity of actual experimental data. The data, obtained from an open dataset [225], contain a human respiratory signal while the participant watched a scary video. The respiratory signal is the temporal change in chest dimension associated with the expansion and contraction of the chest cavity, which was measured using a Hall effect sensor placed high on the torso. **a** The temporal change in chest dimension, sampled at 10 Hz, was used as the x variable for attractor reconstruction (**b**) and to generate the recurrence plot (**c**). **b** Three-dimensional plots of an attractor reconstructed using delay-time embedding ($\tau =0.6\,\mathrm{ s})$. Variables y and z represent respiratory signals 0.6 s and 1.2 s advanced relative to the x variable, respectively. **c** Recurrence plot of human respiration while the participant watched a scary video ($m=3, \tau =0.6\,\mathrm{ s}$, *r* = 0.8). **d** The log–log plot of a distance r versus the correlation sum C(r) has a linear scaling region, indicative of a fractal property. **e** The slope of the log–log plot ($\frac{\mathrm{log}(C\left(r\right))}{\mathrm{log}(r)}$) converges to 1.5 as the embedding dimension grows

Attractors that originate from complex systems often exhibit *fractal structure* or *self-similarity*, where similar patterns appear at increasingly small scales. A widely used index for characterizing fractal structures is the *correlation dimension*, which was proposed by Grassberger and Procaccia [18]. For any positive number *r* and embedding dimension *m*, the correlation sum $C\left(r\right)$, which is the discrete version of the correlation integral, is defined as the fraction of pairs in the time delay embedding vector ${X}_{i}^{m}$ that have distances smaller than *r*,

$${C}_{m}(r)=\frac{1}{N\mathrm{^{\prime}}}\sum_{i=1}^{N\mathrm{^{\prime}}}\sum_{j=(i+1)}^{N\mathrm{^{\prime}}}\Theta \left(r-\Vert {X}_{i}^{m}-{X}_{j}^{m}\Vert \right),$$

where ${N}^{^{\prime}}=N-(m-1)\tau$, $\Vert \cdot \Vert$ is the Euclidian norm, and $\Theta$ is the Heaviside function [19]. If an attractor reconstructed by delay-time embedding exhibits a fractal structure, there exists a region in $r$ where $\frac{\mathrm{log}(C\left(r\right))}{\mathrm{log}(r)}$ is linear. The correlation dimension is defined as the slope of this scaling region. Figure 2 shows the attractor reconstruction, recurrence plot, and correlation dimension estimation from an actual respiratory signal.

The amount of information and the unpredictability of fluctuations in time-series data can be calculated directly using another entropy measure, the *approximate entropy* (ApEn) [9, 20]. The algorithm first estimates the appearance frequency of a similar pattern of sequences by evaluating whether a sequence of data points with length *m* is similar to other sequences in the data, with an allowed distance of $r$ between the points. For each sequence ${X}_{i}^{m}, i=1, \dots , N-m+1$, the appearance frequency is defined as

$${C}_{i}^{m}\left(r\right)=\frac{1}{N-m+1}\sum_{j=1}^{N-m+1}\Theta \left(r-\Vert {X}_{i}^{m}-{X}_{j}^{m}\Vert \right)$$

where *m* is the length of the sequence, *N* is the number of data points, $\Vert \cdot \Vert$ is the norm, and $\Theta$ is the Heaviside function. Then, ApEn can be measured using the average logarithmic appearance frequency:

$${\Phi }^{m}\left(r\right)=\frac{1}{N-m+1}\sum_{\mathrm{i}=1}^{N-m+1}\mathrm{log}({C}_{i}^{m}\left(r\right))$$

$$\mathrm{ApEn}={\Phi }^{m}\left(r\right)-{\Phi }^{m+1}\left(r\right)$$

The *sample entropy* (SampEn) is a modification of the ApEn that is used to assess the complexity of physiological time-series signals and to diagnose disease states [21]. SampEn is defined as

$$\mathrm{SampEn}= -\mathrm{ln}\frac{{{C}^{^{\prime}}}_{m+1}\left(r\right)}{{{C}^{^{\prime}}}_{m}\left(r\right)}$$

$${{C}^{^{\prime}}}_{m}\left(r\right)=\sum_{i=1}^{N-m+1}\sum_{j=\left(i+1\right)}^{N-m+1}\Theta \left(r-\Vert {X}_{i}^{m}-{X}_{j}^{m}\Vert \right)$$

Correlated variability arises from either a stochastic process (e.g., Brownian motion) or a deterministic nonlinear process. Due to various measurement restrictions, respiratory data have a limited length. As a result, any nonlinear dynamics that originate from deterministic processes cannot be distinguished from correlated variations caused by stochastic events [22]. To detect nonlinearity in the observed data, the *surrogate test* has often been used. In this test, surrogate data are generated based on a model or a combination of Fourier and inverse Fourier transformations. The surrogate data share the same statistical properties, such as the AR and power spectrum, as the original data, but do not retain their nonlinear properties [23]. Then, a null hypothesis against the presence of nonlinearity is tested using Monte Carlo methods [23, 24]. Namely, a discriminating statistic of nonlinearity is calculated for the original and all surrogate data, and the null hypothesis is rejected if the value for the original dataset is significantly different from that of the surrogate dataset.

A major obstacle for detecting the nonlinearity of respiratory variability is that nonlinear characterization methods, such as Lyapunov exponents and correlation analyses, are sensitive to both uncorrelated and correlated noise [25]. In addition, although the surrogate test infers the presence of nonlinearity, the results are not sufficient for determining the presence of chaos [26]. An alternative method that can circumvent these limitations is *noise titration*. In this method, noise is gradually added to the observed data by increasing the standard deviation of the data. The noise limit (NL) is defined as the standard deviation at which nonlinearity cannot be detected by the Volterra–Wiener algorithm, with NL > 0 indicating the presence of chaos [25]. There are some circumstances in which noise titration fails to distinguish colored noise from chaos; however, a ‘remedy’ has been provided to address these circumstances [27].

## Sources of respiratory variability

The respiratory control system is complex; while the primary goal of the respiratory system is to maintain arterial blood gas homeostasis, if the work of breathing is too costly, the homeostatic maintenance function can be compromised, and the magnitude and pattern of the respiratory motor output are optimized in terms of a cost function [28, 29]. Swallowing, coughing, sighing, and other nonrespiratory motor acts reset the respiratory rhythm produced by the respiratory central pattern generator (rCPG) in the brainstem. Respiratory neurons in the Bötzinger complex and ventral respiratory group are involved in generating the spatiotemporally organized activities associated with coughing and swallowing, and some respiratory neuronal networks are shared by nonrespiratory networks [30, 31]. Spatial network reorganization, i.e., the expansion and contraction of the active network during the inspiratory phase of breathing, occurs within the rCPG via a balance between excitation and inhibition [32]. This network sharing and reorganization contribute to the flexibility and variability of breathing [33, 34]. Furthermore, volitional and emotional controls of breathing can take control over the pattern of breathing, either consciously or unconsciously, via direct projections to respiratory motoneurons and projections to diverse respiratory control areas in the midbrain, pons, and medulla oblongata [35–38]. This multifaceted control produces variations in breathing that are not random but have some structure inherited from past breaths. In this section, sources of respiratory variability at each level—from automatic control by respiratory rhythmic cores in the brainstem to respiratory control by cortical and subcortical systems—are reviewed.

### Complex variability intrinsic to the rCPG

The characteristics of a given breath were found to be dependent on the characteristics of the immediately preceding breath in paralyzed, artificially ventilated, and vagotomized cats whose spinal cords were cut at the T1 level [39]. This suggests that the nonrandom variability of breathing originates at least in part in the rCPG. The pre-Bötzinger complex (preBötC), which is located in the ventrolateral medulla, is the respiratory rhythmic core that generates the bursting activity that triggers inspiration [40–42]. Another respiratory oscillator, the parafacial respiratory group (pFRG, also known as the lateral parafacial), which is located ventrolateral to the facial nucleus, is an expiratory rhythm generator that causes active expiration during states of elevated respiratory drive [40, 43, 44]. Neurons in the preBötC autonomously exhibit periodic bursting activity due to their channel properties even if they are isolated and produce periodic inspiration-related synchronized activity in slice preparations [45, 46].

Del Negro et al. [47] analyzed respiratory variability at the neuron population level in a highly reduced, but rhythmically active preparation by measuring the integrated preBötC activity. A *Poincaré map*, which is an intersection of the state space, was obtained by plotting $\int {PBC}_{n+1}$ against $\int {PBC}_{n}$, where $\int {PBC}_{n}$ represents the n-th integrated preBötC activity. They observed discrete transitions in the Poincaré maps from periodic oscillations to mixed-mode periodicity, quasiperiodicity, and finally disorganized aperiodic activity, with progressive increases in neuronal excitability, suggesting that the preBötC produces neural activity characteristic of a nonlinear dynamic system. Koshiya et al. [48] applied a voltage-sensitive dye imaging technique to rhythmically active slices and recorded spatiotemporal preBötC activity. They observed that the center of activity, which is calculated according to the magnitude of fluorescence intensity, moved within the preBötC during neuronal population bursts. The state space reconstructed from the moving speed of the center of activity was quasiperiodic, and a correlation dimension analysis and surrogate test suggested the presence of nonlinear dynamics.

Carroll and Ramirez [49] investigated cycle-to-cycle variability during preBötC neuronal recruitment using a multielectrode recording technique and found that respiratory neurons were stochastically activated with each burst. Furthermore, they found that the burst onset variability could not be reproduced in fully interconnected computational models but could be reproduced in sparsely connected network models with as little as 1% connectivity. However, this estimate was based on a randomly connected network that included only excitatory neurons; the preBötC neuronal network includes both excitatory and inhibitory neurons [50, 51]. A higher burst onset variability may be achieved with a higher fraction of all-to-all synaptic connections in a more realistic network with both excitatory and inhibitory neurons since the interburst interval becomes variable when inhibitory connections are included [52].

The pFRG was first identified as a presumptive rhythm generator that triggers the inspiratory pattern generator [53] in the brainstem–spinal cord preparations of neonatal rodents developed by Suzue [54]. The pFRG partially overlaps with the retrotrapezoid nucleus (RTN), which is located ventromedial to the facial nucleus, contains chemosensitive cells and distributes a CO_2_-dependent excitatory drive to the respiratory network [45, 55–57]. In more mature, intact preparations, neurons in the pFRG appear to be quiescent; however, they generate late-expiratory bursts of action potentials when they are disinhibited or activated [58]. Therefore, the pFRG is postulated to be a conditional oscillator for active expiration [40, 43]. Neurons in the preBötC and pFRG are bidirectionally connected; therefore, these inspiratory and expiratory oscillators are coupled. When the excitability of the preBötC network decreases, the inspiratory bursts skip their expected timings in an unpredictable manner; thus, *quantal slowing* of the respiratory rhythm, a phenomenon in which the respiratory rhythm jumps nondeterministically to integer multiples of the control period, occurs [59]. This quantal slowing could be caused by transmission failure from the pFRG to preBötC networks due to suppressed or stochastic excitatory synaptic transmission [59, 60]. Alternatively, quantal slowing could result from a breakdown of synchronized bursting in the preBötC [61]. In either case, the coupled oscillators in the preBötC and pFRG produce respiratory variability similar to atrioventricular blocks in the heart.

Normal breathing consists of three phases: inspiration, postinspiration, and late expiration, all of which are believed to originate in the rCPG in the brainstem [41, 62]. Sequential transection experiments have shown that the three-phase rhythm requires the integrity of the pontine–medullary respiratory network [63]. The pons plays two major roles in the rCPG, with both mediated by neuronal circuitry within the Kölliker-Fuse (KF) area [64]. First, the pons provides an inspiratory off-switch that causes an inspiratory-to-expiratory phase transition in conjunction with sensory feedback from slowly adapting pulmonary stretch receptors. Second, the pons regulates postinspiration, adjusting upper airway resistance during the respiratory cycle. Lesioning the pons results in a longer and more irregular inspiratory phase [65–67]. Stimulus after-effects (the prolongation of the inspiratory duration) have been found to be augmented following lesioning, suggesting that the pons plays an important role in the stability of the rCPG [67].

Yu et al. [68] investigated the effects of changing the input to the pons using a conductance-based model of the four different types of cells in the rCPG. The model shows that reduced pontine input causes longer inspiratory phases, reduced respiratory rate (RR), and increased breath-to-breath variability, consistent with the experimental findings. Furthermore, they investigated how channel noise affects neural dynamics at the circuit level. The model predicted that the expiratory phase is more variable than the inspiratory phase when the channel number is small, and vice versa when the channel number is large. Among the four different types of cells, the pacemaker cell exhibited the highest sensitivity to channel noise.

### Astrocytic contributions to respiratory variability

Astrocytes respond to changes in neuronal network activity across brain states and behaviors [69] and modulate central pattern-generating motor circuits [70]. Astrocytes can even modulate brain-wide oscillations by transmitting the oxygenation status to higher cortical areas [69]. In the preBötC, the astrocyte glutamate–glutamine cycle and the supply of glutamine to neuronal glutamatergic terminals are essential for rhythm generation [71]. Furthermore, blocking the vesicular release of preBötC astrocytes reduces the resting breathing rate, lowers the frequency of periodic sighs, decreases rhythm variability, impairs respiratory responses to hypoxia and hypercapnia, and reduces exercise capacity [72].

**Fig. 3:**
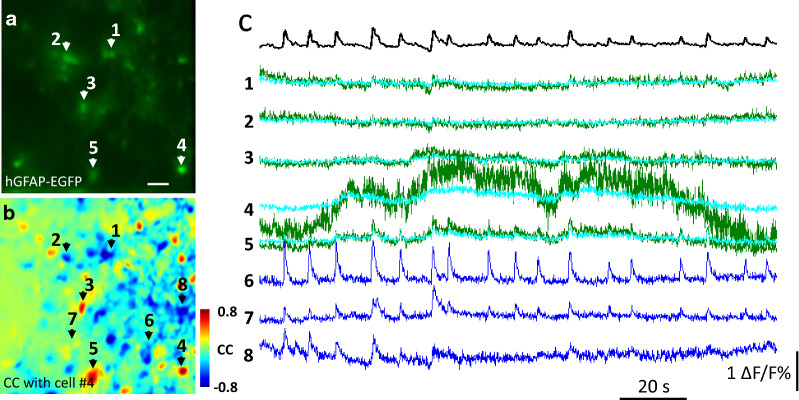
Representative calcium transients of EGFP-positive and EGFP-negative cells [79]. **a** Astrocytes are identified with GFAP promoter-controlled expression of EGFP. Scale bar: 40 µm. **b** Cross-correlation image between each pixel and the low-frequency calcium oscillation of cell #4 indicates that low-frequency astrocytic calcium oscillations are highly synchronized. **c** The top trace shows the respiratory rhythmic calcium fluctuation, and the other traces show bandpass filtered calcium signals of representative cells. For cells #1–8, the location of each cell is indicated in the left panels (**a** and **b**). For EGFP-positive cells (#1–5), the calcium signals of the cell (green) and its vicinity (light blue) are shown

**Fig. 4:**
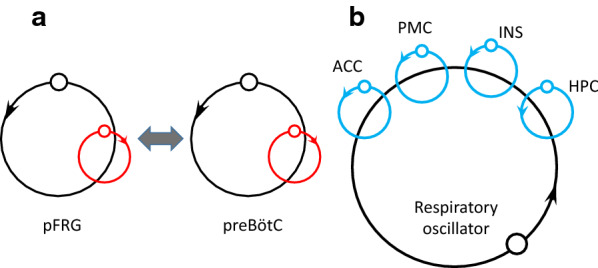
Schematic drawings of coupled slow and fast oscillators that exhibit cross-frequency coupling (CFC). **a** Slow glial (red circles) and fast neuronal (black circles) oscillators are organized within the pFRG and preBötC and modulate their intrinsic rhythms [75]. The pFRG and preBötC conditionally couple, resulting in complex variability, such as quantal slowing. **b** The slow respiratory oscillator (black circle) orchestrates fast neuronal oscillations (blue circles) in the anterior cingulate, premotor, insular, and hippocampal cortices via CFC [151, 152]. *pFRG* parafacial respiratory group, *preBötC* pre-Bötzinger complex, *ACC* anterior cingulate cortex, *PMC* premotor cortex, *INS* insular cortex, *HPC* hippocampal cortex

Rhythmic, inwardly directed currents attributed to neuronal population bursts have been found in 10% of preBötC astrocytes [73]. Okada et al. [74] recorded the spatiotemporal activities of neurons and astrocytes in the preBötC using a calcium imaging technique. They found that a subset of astrocytes exhibited preinspiratory increases in the intracellular calcium concentration that were irregularly coupled with inspiratory neuronal bursts. In addition, they found that optogenetic stimulation of the astrocytes triggered action potentials in inspiratory neurons in the preBötC. Similar irregular coupling between the calcium activities of neurons and astrocytes has been reported in organotypic cultures of preBötC slices and pFRG slices [75, 76]. Network structure analyses with the cross-correlation technique and graph theory [77] revealed three separate but interconnected subnetworks: the glial, neuronal, and glial-neuronal networks [76]. These networks are organized into a small-world network structure commonly observed in biological networks [78]. On the other hand, half of the preBötC astrocytes showed synchronized low-frequency (0.023 Hz) oscillations; thus, a subset of the astrocytes forms a slow oscillator (Fig. 3) [73, 74, 79]. Therefore, the neurons and astrocytes in the preBötC and pFRG may form coupled slow and fast oscillators and can mutually interact, thus producing complex behaviors (Fig. 4a) [79, 80].

**Fig. 5:**
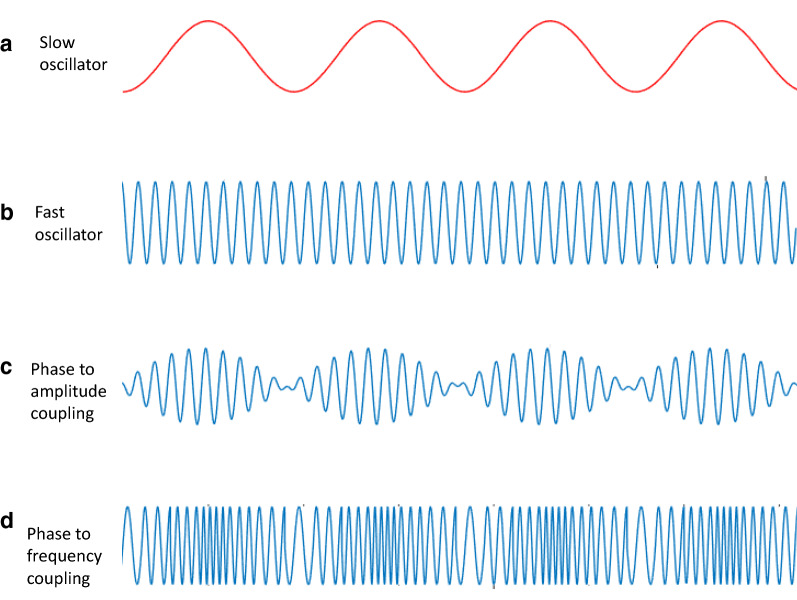
Representative modes of cross frequency interactions. Consider a slow oscillator (**a**) that interacts with a fast oscillator (**b**). In **c**, the power of the fast oscillation is modulated by the phase of the slow oscillation. This mode of interaction can be observed between the phase of respiration (slow oscillation) and the power of the gamma-band (40–150 Hz) oscillations during attention to breathing [151], and between the phase of respiration and the power of alpha-band (8–13 Hz) oscillations during a near-threshold spatial detection task [147]. In **d**, the frequency of the fast oscillation is modulated by the phase of the slow oscillation

Coupled oscillators desynchronize for sufficiently small couplings and then bifurcate to partially synchronized states when the coupling increases above a critical value [81]. *Cross-frequency coupling* (CFC), which is the interaction between oscillations in different frequency bands, is a widely observed phenomenon in the brain that may play a functional role in neuronal computation, communication, and learning [82]. There are various types of CFC, including phase-phase, phase-frequency, phase-amplitude, amplitude-amplitude, frequency-frequency, and amplitude-frequency (Fig. 5) [83]. In particular, phase-amplitude coupling changes quickly in response to sensory, motor, and cognitive events and correlates with performance in learning tasks (see "Control of respiration during cognition" section) [82]. However, the coupling between glial and neuronal oscillators may be more complex, since the coupling is not compatible with any known type of CFC. Therefore, this interaction may be revealed only through nonlinear analyses, such as cross-recurrence plot analysis [84, 85].

### Correlated variability and oscillations originating from the chemical control system

The integrated phrenic nerve activity ($\int \mathrm{Phr}$) responses to the electrical stimulation of a carotid sinus nerve (CSN) in anesthetized, paralyzed, vagotomized, and glomectomized cats have two distinct components: a rapid increase that accounts for approximately half of the full response and a gradual increase that eventually reaches a steady-state plateau [86]. After the stimulation stops, *short-term potentiation* (STP) of respiration is observed, in which the level of $\int \mathrm{Phr}$ decreases rapidly but remains higher than the prestimulation level [86]. STP has also been observed after acute exposure to hypoxia, which is known as *post-hypoxic persistent respiratory augmentation* (PHRA) [87, 88]. Arundic acid, which is an astrocyte inhibitor, has been shown to suppress PHRA; however, astrocyte-specific Trpa1 knockout did not abolish PHRA, indicating that astrocytes mediate PHRA through mechanisms other than the putative ventilatory hypoxia sensor TRPA1 channels [89].

In addition to STP, several different types of memory effects have been shown to be associated with chemoreceptor afferent activation [90, 91]. CSN stimulation or hypoxia exposure increases $\int \mathrm{Phr}$; however, the RR is below that of the steady-state baseline level. This phenomenon is known as the *short-term depression* (STD) of respiration. Injections of muscimol, a GABA_A_ agonist, into the ventrolateral pons, where low current pulses evoke short-latency inhibition of phrenic nerve activity, have been shown to abolish STD [92]. This result suggests that the integrity of the ventrolateral pons is required for STD [92]. While STD involves a decrease in RR that occurs when hypoxic exposure is sustained for tens of seconds to a few minutes, *hypoxic ventilatory depression* (HVD) involves a decrease in tidal volume (VT) that occurs when moderate hypoxemia is sustained for 5–30 min [90]. Although the mechanism of HVD is unknown, recent findings have shown that astrocytes in the preBötC play an important role in counteracting HVD by releasing ATP to stimulate ventilation by activating P2Y_1_ receptors [93]. Repeated CSN stimulation or hypoxia exposure has been shown to result in *long-term potentiation* (LTP) of respiration in rats, where $\int \mathrm{Phr}$ remained above that of the controls for at least 30 min. Vermalectomy eliminated LTP, which suggests that the cerebellar vermis plays a role in LTP [91].

The above experiments on STP, STD, and LTP were all conducted under open loop conditions, i.e., without chemical feedback control; therefore, these memory effects must be caused by neural mechanisms rather than chemical feedback mechanisms. However, correlated respiratory activity can also arise from chemical feedback [1, 94]. The VT of each breath in anesthetized, vagotomized, and spontaneously breathing rats was correlated with that immediately preceding breathing; however, such a correlation was not observed in the $\int \mathrm{Phr}$ of anesthetized, vagotomized, paralyzed, and artificially ventilated rats [94]. Therefore, the autocorrelation originates from chemical feedback mechanisms.

Hypoxia, in combination with sleep and hypocapnia, can induce periodic breathing characterized by repeated clusters of two to five breaths interspaced with regularly spaced expiratory pauses [95]. During wakefulness, isocapnic hypoxia increases gross respiratory variability but decreases the autocorrelation coefficient at a lag of one breath for minute ventilation. The increase in respiratory variability can be decomposed into a random component and an oscillatory component, indicating that hypoxia induces hidden oscillations even in the absence of hypocapnia in healthy awake subjects [96]. The PaCO_2_ level also affects respiratory variability. Hypercapnia reduces respiratory variability and increases LLE, whereas hypocapnia increases respiratory variability and decreases LLE [97].

### Phase resetting of the respiratory rhythm

The response dynamics of chemical feedback and mechanoreceptor feedback systems differ, with the latter depending on when the stimulus is presented [98]. For example, lung inflation during inspiration shortens the inspiratory time, whereas lung inflation during expiration prolongs the expiratory time. This suggests that the predicted timing of a certain phase, e.g., the onset of inspiration, can shift depending on the timing of a stimulus. This phenomenon is referred to as the *phase resetting* characteristic [99]. The phase resetting characteristics of the rCPG were first investigated by electrically stimulating the midbrain [100], which facilitates inspiration; however, these characteristics were most extensively studied by electrically stimulating the vagus nerve and superior laryngeal nerve (SLN) [1], which suppress inspiratory activity during most phases of the respiratory cycle. A plot of the time between the onset of the preceding inspiration and a stimulus (termed the *old phase*) versus the time between the stimulus and the onset of the following inspiration (termed the *cophase*) determines the topographical type of phase resetting [100]. If the stimulus is weak, a net change in the cophase as the old phase moves through one respiratory cycle becomes one respiratory cycle, and the topographical type is classified as *type-1*. If the stimulus is strong, the net change becomes 0, and the phase resetting is classified as *type-0*.

A brief SLN stimulation produces after-effects that can last for several cycles [101]. The stimulus after-effects depend on the timing of a given stimulus. Brief vagal nerve stimulation delivered during each respiratory cycle at mid-inspiration, mid-expiration, and late expiration near the expiratory-to-inspiratory phase transition can result in complex breath-to-breath variability [102]. Dhingra et al. [103] investigated vagal-dependent respiratory variability using information theory-based techniques and surrogate data testing. They found that the vagal afferent and dorsolateral pons both contribute to nonlinear variability in the pattern of breathing and are mutually dependent.

Repeated stimulation entrains respiration to the stimulation; however, the trajectories that return to the baseline state are not the same, resulting in respiratory variability. The stochastic noise associated with ion channel gating and synaptic neurotransmission affects the entrainment of respiratory rhythms to external periodic inputs [104]. Periodic vagal nerve stimulation (VNS) entrains the respiratory rhythm similar to the Hering-Breuer reflex; however, VNS increases respiratory variability because noise interacts with the input, leading to *phase slips*. Suppressing the activity of the KF region enhances entrainment and reduces rhythm variability during VNS, which suggests that the KF region regulates respiratory variability by controlling the gain of the Hering-Breuer reflex [104].

### Swallowing

Swallowing is a physiological perturbation of the respiratory rhythm [105, 106]. In humans, swallowing during inspiration terminates the inspiratory phase, and respiration resumes with expiration, with a shorter duration than that of the control [107]. On the other hand, swallowing during expiration interrupts the expiratory phase; however, in this case, respiration normally resumes with expiration, increasing the total duration of the expiratory phase [107]. In general, swallowing acts as a strong perturbation in the rCPG, resulting in type-0 phase resetting [106, 107]. All rCPG neurons appear to be affected by swallowing, irrespective of the type of neuron. Inspiratory-augmenting, inspiratory-decrementing, and expiratory-augmenting neurons are all inhibited [31, 108, 109], while a majority of expiratory-decrementing neurons are activated in rats [108], and inspiratory-to-expiratory phase-spanning neurons are activated in guinea pigs [109]. Lesioning the KF area with ibotenic acid eliminates the respiratory phase resetting caused by swallowing, which suggests that the KF region plays an important role in coordinating breathing and swallowing [110, 111]. Furthermore, KF inhibition attenuates tonic postinspiratory vagal nerve activity and lowers the threshold for evoking swallowing. Therefore, the KF region plays a role in the airway-defensive laryngeal adductor reflex and gates the initiation of swallowing [112]. Since swallowing initiation is also inhibited by vagal feedback, dual peripheral and central gating mechanisms are involved in the coordination between breathing and swallowing [113].

### Sighing

Sighs originate from a small ensemble of preBötC neurons [33, 114]. Physiological sighing requires peptidergic inputs from RTN/pFRG neurons, which express the bombesin-like neuropeptide neuromedin B or gastrin-releasing peptides [40, 114]. Physiological sighing is believed to be important in preventing the alveoli from collapsing (atelectasis), improving gas exchange [115, 116], and reducing hypoxia and hypercapnia [117]. However, a sigh is not only an augmented breath that maximally inflates the lung but also signals brain state changes, controls arousal, and regulates homeostasis of respiratory variability [118]. Vlemincx et al. [119] found that sighing increases autocorrelated respiratory variability and relieves mental stress. They hypothesized that sighs serve as psychophysiological resetters, restoring respiratory regulation by resetting the nonrandom respiratory variability when it becomes too low or too random [120–122]. Based on the theory of stochastic resonance [123], they postulated that an inappropriate level of respiratory variability compromises flexible and adaptive responsiveness or jeopardizes stability and hypothesized that a sigh acts as noise to restore healthy balanced respiratory variability [119]. Meanwhile, because autocorrelated respiratory variability arises from chemical feedback control [1, 94], the level of autocorrelated variability may reflect the relative contribution of the chemical feedback control to the total respiratory variability. Thus, sighing may shift respiratory control from the cortical and subcortical systems to the brainstem autonomic control system.

### Breathing controlled by the limbic system

Emotion induces various physiological responses, including changes in heart rate, blood pressure, body temperature, and respiratory patterns, by activating the autonomic nervous system [124]. Among the changes in respiratory patterns, changes in the RR have been investigated extensively. Negative emotions, such as anxiety [125], fear [126], and sadness [127] increase the RR. Positive emotions, such as happiness, increase the RR [126, 127], while relief decreases the RR [125]. In addition to changes in the RR, each emotion appears to accompany a characteristic pattern of breathing that, to some extent, may overlap with the pattern from other emotions [128]. For example, fast, deep breaths are associated with excitement, while rapid, shallow breaths are associated with concentration, fear, and panic. Discriminant analyses indicate that four emotions (anger, fear, happiness, and sadness) can be adequately classified using heart rate variability, respiratory sinus arrhythmia, the mean RR, and respiratory variability, suggesting that distinct patterns of peripheral physiological activity are associated with different emotions [126].

The response to an emotion in the respiratory pattern is affected by the personality of the subject [129]. In subjects with high levels of anxiety, increases in the RR are more dominant responses to mental stress than changes in the VT [130], and changes in expiratory duration are dependent on anxiety scores [131]. Personality even affects respiratory parameters of subjects at rest [132]. The VT is smaller and the RR is higher in subjects with more anxiety and higher states on the State-Trait Anxiety Inventory [132].

The RR increases during the interval between alert presentation and actual stimulation, irrespective of changes in O_2_ consumption [133], which implies that breathing is controlled by the cortical or subcortical areas associated with emotion during the anticipatory anxiety period. Masaoka et al. [134] analyzed electroencephalogram (EEG) data during the anticipatory anxiety period and observed positive waves in cycle-triggered averaged EEG signals approximately 350 ms after the onset of inspiration, which are known as respiratory-related anxiety potentials (RAPs). A dipole tracing analysis based on a scalp-skull-brain head model identified the source location of the RAPs as the right temporal pole, while in the most anxious subject, it was the temporal pole and the amygdala [134]. A blood oxygen level-dependent (BOLD) functional magnetic resonance imaging (fMRI) study [135] demonstrated that the insula is essential for dyspnea perception. In addition, activation of the anterior cingulate cortex was correlated with the Breathlessness Catastrophizing Scale during dyspnea anticipation [136].

The respiration–emotion relationship is bidirectional. Deep and slow breathing (DSB) reduces anxiety and skin conductance levels in alcohol-dependent young adults [137]. Older adults also benefit from DSB in terms of vagal tone and anxiety [138]. The ameliorating effects of DSB on anxiety are believed to be mediated by reinforcement of the vagal tone, which balances sympathetic and parasympathetic activity [139]. Philippot and Blairy [128] tested whether respiratory changes affect emotions. When subjects mimicked a breathing pattern characteristic of joy, anger, fear, or sadness, the emotional state characterized by that breathing pattern was evoked, suggesting that alterations in breathing patterns can induce emotion. Masaoka et al. [140] showed that odors associated with autobiographical memories can trigger DSB and pleasant emotional experiences.

### Control of respiration during cognition

Grassmann et al. [141] conducted a systematic review on respiration and cognitive loads. They found that in general, the cognitive load increases the RR and minute ventilation while not considerably impacting the VT. The end-tidal CO_2_ level decreased, which suggests that subjects were hyperventilated; however, oxygen consumption and CO_2_ release were also elevated. Changes in respiratory variability depend on the type of cognitive load [142]. Total variability in the RR decreases during sustained attention tasks, while during an arithmetic load, the autocorrelated variability decreases while the random variability increases. In addition, the frequency of sighing increased *during* sustained attention tasks but *after* arithmetic tasks, suggesting that the need for the respiratory control system to reset differs depending on the type of load [142]. Honma et al. [143] found that, compared with reading on paper, reading on a smartphone elicited fewer sighs and promoted brain overactivity in the prefrontal cortex, resulting in reduced comprehension.

There is growing evidence that human subjects can adjust their respiratory cycle to the onset of cognitive tasks, even if the tasks are not olfactory in nature [144–148]. Johannknecht et al. [146] found that subjects tend to align their respiratory cycle to the experimental paradigm, inhaling when the stimulus is presented and exhaling when submitting their responses. Respiratory timing affects cognitive task performance [145, 146, 148–150]. Zelano et al. [150] recorded intracranial EEG (iEEG) signals in patients with epilepsy and found that natural breathing synchronized electrical activity in the pyriform cortex, amygdala, and hippocampus. Fear discrimination and memory retrieval were enhanced during the inspiratory phase when the oscillatory power peaked. Cognitive performance was modulated during nose breathing but not during mouth breathing. Furthermore, Herrero et al. [151] demonstrated that coherence between the iEEG signal and breathing increased in the frontotemporal-insular network during volitionally paced breathing, whereas attention to breathing increased coherence in the anterior cingulate, premotor, insular, and hippocampal cortices. They proposed that breathing can organize neuronal oscillations throughout the brain [151]. In addition, Kluger et al. [152] applied phase-amplitude analysis to magnetoencephalography (MEG) data from quiet breathing humans and demonstrated the presence of respiration-mediated CFCs, termed respiration-modulated brain oscillations, across all major frequency bands in a widespread network of cortical and subcortical areas (Fig. 4b). Furthermore, they showed that occipital alpha power was coupled with respiration during near-threshold spatial detection tasks and that this respiration-alpha coupling was maximized with a respiratory phase lag of − 30°, indicating that the coupling occurs before behavioral consequences [147]. Time–frequency analyses revealed that compared with the alpha power prior to the presentation of undetected targets, the alpha power prior to the presentation of detected targets was significantly suppressed. Based on these results and the ‘active sensing’ concept for the functional role of olfaction [153], Kluger et al. [147] suggested that respiration actively adjusts the timing of sensory information sampling with transient oscillatory cycles of heightened cortical excitability to optimize performance. These respiratory acts are conceivably regulated by higher brain networks, albeit unconsciously; however, it has been shown that a subset of preBötC neurons regulates the balance between calm and arousal behaviors in a bottom-up fashion [154].

## Respiratory variability in health and disease

Goldberger [4] presented an innovative concept: the output of a ‘healthy’ control system is not constant but instead fluctuates in a complex manner. Techniques that measure the complexity of the output have indicated that physiological control systems operate far from equilibrium and that maintaining constancy is not the goal. For example, fluctuations in the heart rate of healthy humans are chaotic [155] and multifractal, as they can be decomposed into multiple scaling regions [156], whereas fluctuations in the heart rates of patients with chronic heart failure (CHF) are less chaotic [155] and monofractal [156]. Aging also decreases the complexity of heart rate fluctuations, as quantified by SampEn [157]. Based on these observations, one might predict that the complex variability in respiratory signals would be greater in healthy individuals than in individuals with a disease. While this prediction is true in some cases, this is not always the case.

In human neonates with mild respiratory distress syndrome, the RR and VT exhibited increased complexity with increasing weight and gestational age; however, this complexity was observed only in terms of pattern matching-based entropies and not in the ApEn and SampEn, which are based on conditional probabilities [158]. This implies that respiratory fluctuations become increasingly complex with maturation. On the other hand, the LTC in the interbreath interval (IBI) time series decreases with age [159], similar to the changes in the complex variability of the heart rate [157]. Furthermore, sex affects changes in respiratory complex variability over the course of aging. The scaling exponents of the IBI time series are significantly lower (indicating decreased correlations) in healthy older males than in young males, young females, and older females [159]. The correlation dimensions of respiratory movement are lowest during slow wave sleep (stage IV) and highest during rapid eye movement (REM) sleep, with both correlated with the correlation dimension of the EEG signals [160]. STCs in the VT and minute ventilation, which may indicate the chemical control of breathing, have been observed during both non-REM and REM sleep, while LTCs have been observed only during REM sleep [12]. Exercise has opposite effects on the complex variability of respiration and heart rate, inducing a decrease in STCs in the IBI and an increase in LTCs of heart rate variability [161].

Various diseases affect respiratory variability. Breathing variability is remarkably augmented in patients with anxiety disorders, such as panic disorder [162–165]. Ventilatory complexity is also increased in patients with hyperventilation disorder; however, their respiratory control stability, which is assessed based on the loop gain, is not impaired [166]. In patients with breathing pattern disorders, a prevalent cause of exertional dyspnea, the ApEn of the VT and minute ventilation during the cardiopulmonary exercise test was significantly greater than that of controls [167].

The ventilatory flow of healthy, quietly breathing subjects exhibits nonlinear dynamics that are indicative of chaos [26]. In critically ill patients, switching from assist-controlled mechanical ventilation to inspiratory pressure support reduced the CV and eliminated nonlinear dynamics that are detectable using the noise titration technique [168]. Therefore, the chaotic feature of respiratory variability is neurogenic and is either intrinsic to the rCPG, a result of respiratory control processes driven by perturbations, or both, with little contribution from lung mechanics, if any [168]. However, changes in lung mechanics and gas exchange affect the gross variability [169, 170] and complexity of respiratory fluctuations [171–173]. The VT, RR, and minute ventilation are greater and the CV of the inspiratory time and minute ventilation are lower in patients with chronic obstructive pulmonary disease (COPD) than in healthy controls [170]. The random fraction of the breath variability is reduced, and the nonrandom, correlated fraction is greater in patients with restrictive lung disease than in healthy controls [169]. Interestingly, small variations in the average resting VT led to marked increases in dyspnea in these patients [169]. Compared with healthy subjects, the ApEn was significantly reduced in patients with asthma, which was correlated with the spirometric indices of airway obstruction [173]. Furthermore, the SampEn was significantly reduced in patients with COPD, which was also correlated with the spirometric indices of airway obstruction [171]. In addition, acute bronchodilation increased ventilatory complexity, as quantified by the noise titration technique during resting breathing in patients with stable COPD [172]. These changes in respiratory variability in patients with lung diseases may be a direct consequence of feedback from chemoreceptors and mechanoreceptors in the lung and airway. Alternatively, these changes could be due to alterations in autonomic function. Sympathetic nerve activity is increased in patients with COPD [174, 175], which is associated with morbidity and mortality [176]. Sympathetic neural overactivity may be a consequence of chronic hypoxia exposure [177]; however, slow breathing reduces elevated sympathetic activity in patients with COPD [178].

The most remarkable form of respiratory variability caused by instability in the chemical feedback loop is a type of periodic breathing known as *Cheyne-Stokes respiration (CSR)*. CSR is a specific form of central sleep apnea characterized by the waxing and waning of the VT in 50 ~ 90 s intervals [179]. CSR has been observed in patients with CHF, particularly during stages 1 and 2 of non-REM sleep [180]. CSR has also been observed during the day and is more closely correlated with the severity of CHF [181]. The mechanism underlying CSR is thought to be a failure in chemical feedback control [179, 182, 183]. In control theory, the stability of a feedback system is defined by the controller gain, the plant gain, and the loop gain (LG). The controller gain is a measure of how much the controller responds to a given change in blood gas tension ($\Delta {\dot{V}}_{E}$/$\Delta \mathrm{Pa{CO}}_{2}$), and the plant gain is a measure of how much the blood gas tension changes for a given change in ventilation ($\Delta {\mathrm{PaCO}}_{2}$/$\Delta {\dot{V}}_{E}$). The LG, which is the product of the controller gain and the plant gain, represents the ratio of the ventilatory response to the ventilatory disturbance. An LG of less than 1 indicates stable breathing, whereas an LG of greater than 1 in combination with a prolonged circulatory delay results in periodic breathing. In CHF patients, a decrease in cardiac output can lead to prolonged circulatory delays and mild hypoxemia, increasing controller gain. These factors, in combination with an increase in plant gain due to sleep hypoventilation and the subsequent elevation of PaCO_2_, destabilize chemical feedback control, resulting in CSR [179]. Moreover, CHF patients tend to hyperventilate and become hypocapnic during wakefulness. Subsequently, the withdrawal of the wakefulness stimulus upon sleep leads to apnea [184, 185]. Nasal continuous positive airway pressure [186, 187] and inhalation of 3% CO_2_ [188] have been shown to ameliorate CSR by reducing plant gain.

Obstructive sleep apnea (OSA) is a common breathing disorder that involves periodic breathing with repetitive narrowing and closing of the upper airway during sleep [189]. The primary cause of OSA is an anatomically collapsible upper airway; however, additional nonanatomical factors, such as inadequate responsiveness of the upper airway dilator muscles during sleep, waking prematurely due to airway narrowing, and a high LG, characterize different phenotypes of OSA [190]. During apnea, both the plant gain and the controller gain increase; thus, the increased LG at the end of the obstruction is not the cause but the result of the obstructive event [191]. The chemical LG measured while the upper airway is stable is moderately elevated in some OSA patients; however, the increase is insufficient for causing instability in the absence of a collapsible upper airway [192, 193].

The breathing pattern of CHF patients is typically characterized by unstable respiration, such as rapid, irregular, and nonperiodic respiration with transient sighing or apnea, rather than CSR [194]. Respiratory instability is unlikely to be related to the negative feedback system of chemical respiratory control; rather, it might be caused by the stimulation of afferent vagal nerve endings due to lung edema [195, 196]. Asanoi et al. [195] developed a quantitative measure of respiratory instability (RSI) based on the frequency distribution of respiratory spectral components and the very low-frequency components. They found that patients who died from cardiac causes had a lower RSI and suggested that an RSI < 20 predicts a higher probability of subsequent all-cause and cardiovascular death. Okamoto et al. [197] analyzed stable airflow data before the onset of sleep to quantify breathing irregularities using the ShEn in patients with relatively mild CHF, ischemic disease, or atrial fibrillation. They found that the ShEn of the airflow signals in these patients was significantly greater than the ShEn of patients without heart disease.

## Future directions in translational sciences

The idea of extracting hidden information from respiratory signals and utilizing these data in clinical practice and daily life is attractive. This process could be easily carried out in intensive care units, where continuous monitoring of breathing is the standard protocol. In intensive care units, respiratory variability may have predictive value for successful weaning from mechanical ventilation [198]. For example, Wysocki et al. [199] showed that the reduced CVs of the TV/inspiratory time and inspiratory time/respiratory period can be used to predict successful weaning cases. Additionally, El-Khatib et al. [200] showed that spontaneous breathing patterns during minimal mechanical ventilatory support are more chaotic in patients who failed extubation trials than in patients who passed them. Similarly, Engoren et al. [201] showed that the RR and ApEn of the VT increase upon spontaneous ventilation in weaning trials for patients who require mechanical ventilation. Nonlinear dynamics analyses can also be used to diagnose specific diseases. Miyata et al. [202] showed that the correlation dimension of chest movement with a brief period during wakefulness may be a useful index for identifying patients with OSA. Raoufy et al. [203] showed that nonlinear analyses (LLE, LTC, and SampEn) of breathing patterns have diagnostic value in asthma and can be used to differentiate uncontrolled and controlled asthma as well as nonatopic and atopic asthma using receiver operating characteristic (ROC) curve analysis.

The gold standard technique for staging sleep is polysomnography; however, this method requires expensive equipment with constrained sensors for recording and human resources for analysis. Therefore, there is a need for an automated sleep staging system that ideally uses an inexpensive, wearable or noncontact sensor. Breathing patterns in infants are considerably different between active sleep (equivalent to adult REM sleep) and quiet sleep (equivalent to adult non-REM sleep) [204]. Haddad et al. [205] reported that the CV of the IBI can be used to adequately distinguish active and quiet sleep stages in newborn infants. Harper et al. [206] applied machine learning techniques to identify sleep stages in newborn infants according to cardiorespiratory variables. Terrill et al. attempted to use nonlinear analyses of respiratory variability for sleep staging [207]. They showed that features extracted from recurrence plots of the IBI using recurrence quantification analysis [17, 208] can be used to classify sleep stages in infants. Recently, machine learning techniques have been applied not only to identify sleep stages but also to detect respiratory events (apnea, hypopnea, and CSR) during sleep [209–211].

Another promising research direction is an application toward emotion recognition. Emotion is tightly coupled with physiological changes that are specific to each emotion (see "Breathing controlled by the limbic system" section) [126, 212]. Advances in wearable sensors for measuring physiological signals and machine learning techniques have allowed e-health research to focus on emotion recognition [213–216]. Emotion recognition technology is expected to be applied in various fields, such as mental health conditioning, man–machine interfaces, marketing, and education [217–221]. Studies on determining human emotions in the engineering field generally use a two-dimensional model known as Russell’s circumplex model of affect for emotion classification [222, 223] since it can easily be used with classification algorithms. The circumplex model assumes that all affective states arise from two fundamental neurophysiological systems: one related to *valence* (a pleasure–displeasure continuum) and another related to *arousal*, or alertness. Although only a few studies have used respiration for emotion recognition to date [214], a study based on deep learning algorithms applied to the dataset DEAP [224] showed valence and arousal accuracies of 73% and 81%, respectively [221].

## Conclusions

Respiratory variability contains a veritable treasure trove of hidden information. The elucidation of the mechanisms underlying this variability is undoubtedly important; however, deep learning techniques and information theory-based quantification of complex variability have allowed us to use this variability for inference and decision making without knowing its precise sources and mechanisms. On the other hand, since the structure of the model is not expected to mimic the actual system in conventional deep learning techniques, the techniques cannot be applied to elucidate the sources and mechanisms of respiratory variability. Rather, these techniques in combination with smart sensors and devices should be used to improve the health and quality of life of everyone.

## Data Availability

Not applicable.

## Abbreviations

CV: Coefficient of variation
RMSSD: Root mean square successive difference
AR: Autocorrelation
LTC: Long-term correlation
STC: Short-term correlation
LLE: Largest Lyapunov exponent
ShEn: Shannon entropy
ApEn: Approximate entropy
SampEn: Sample entropy
NL: Noise limit
rCPG: Respiratory central pattern generator
preBötC: Pre-Bötzinger complex
pFRG: Parafacial respiratory group
RTN: Retrotrapezoid nucleus
KF: Kölliker-Fuse
CFC: Cross-frequency coupling
CSN: Carotid sinus nerve
STP: Short-term potentiation
STD: Short-term depression
LTD: Long-term depression
PHRA: Post-hypoxic persistent respiratory augmentation
HVD: Hypoxic ventilatory depression
SLN: Superior laryngeal nerve
VNS: Vagal nerve stimulation
RR: Respiratory rate
VT: Tidal volume
EEG: Electroencephalogram
RAP: Respiratory-related anxiety potential
BOLD: Blood oxygen level dependent
fMRI: Functional magnetic resonance imaging
DSB: Deep and slow breathing
iEEG: Intracranial electroencephalogram
MEG: Magnetoencephalography
CHF: Chronic heart failure
IBI: Interbreath interval
REM: Rapid eye movement
COPD: Chronic obstructive pulmonary disease
CSR: Cheyne-Stokes respiration
LG: Loop gain
OSA: Obstructive sleep apnea
RSI: Quantitative measure of respiratory instability
ROC: Receiver operating characteristic
